# Circadian‐timed dopamine agonist treatment reverses high‐fat diet‐induced diabetogenic shift in ventromedial hypothalamic glucose sensing

**DOI:** 10.1002/edm2.139

**Published:** 2020-05-07

**Authors:** Carl R. Stoelzel, Yahong Zhang, Anthony H. Cincotta

**Affiliations:** ^1^ VeroScience LLC Tiverton RI USA

**Keywords:** circadian, diabetes, dopamine, insulin sensitivity, ventromedial hypothalamus

## Abstract

**Introduction:**

Within the ventromedial hypothalamus (VMH), glucose inhibitory (GI) neurons sense hypoglycaemia while glucose excitatory (GE) neurons sense hyperglycaemia to initiate counter control mechanisms under normal conditions. However, potential electrophysiological alterations of these two neuronal types in vivo in insulin‐resistant states have never been simultaneously fully documented. Further, the anti‐diabetic effect of dopamine agonism on this VMH system under insulin resistance has not been studied.

**Methods:**

This study examined the impact of a high‐fat diet (HFD) on in vivo electrophysiological recordings from VMH GE and GI neurons and the ability of circadian‐timed dopamine agonist therapy to reverse any adverse effect of the HFD on such VMH activities and peripheral glucose metabolism.

**Results:**

HFD significantly inhibited VMH GE neuronal electrophysiological response to local hyperglycaemia (36.3%) and augmented GI neuronal excitation response to local hypoglycaemia (47.0%). Bromocriptine (dopamine agonist) administration at onset of daily activity (but not during the daily sleep phase) completely reversed both VMH GE and GI neuronal aberrations induced by HFD. Such timed treatment also normalized glucose intolerance and insulin resistance. These VMH and peripheral glucose metabolism effects of circadian‐timed bromocriptine may involve its known effect to reduce elevated VMH noradrenergic activity in insulin‐resistant states as local VMH administration of norepinephrine was observed to significantly inhibit VMH GE neuronal sensing of local hyperglycaemia in insulin‐sensitive animals on regular chow diet (52.4%).

**Conclusions:**

HFD alters VMH glucose sensing in a manner that potentiates hyperglycaemia and this effect on the VMH can be reversed by appropriately circadian‐timed dopamine agonist administration.

## Graphical abstract

This study examined the impact of an obesogenic HFD on in vivo VMH glucose‐sensing mechanisms and the ability of circadian‐timed dopamine agonist therapy to reverse any adverse effect of the HFD on such mechanisms. HFD led to decreased responsiveness of VMH GE neurons to local hyperglycaemia, and an increased responsiveness of VMH GI neurons to local hypoglycaemia. Circadian‐timed bromocriptine administration at the onset of locomotor activity but not during the daily sleep phase reversed both the VMH GE and GI aberrations induced by the HFD.

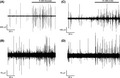

## 1 INTRODUCTION

Daily administration of the dopamine D2 receptor agonist, bromocriptine, for a period of 7‐14 days at a time of day mimics the natural circadian peak of dopaminergic activity in the brain, which is diminished in insulin‐resistant states, to animal models of the insulin‐resistant, glucose‐intolerant state reverses this condition.^1^, ^2^, ^3^, ^4^ Likewise, a quick‐release micronized formulation of bromocriptine (bromocriptine‐QR) similarly administered to humans with type 2 diabetes (T2D) produces similar improvements in postprandial hyperglycaemia^5^ [reviewed in ref. 6]. A series of studies by our laboratory has demonstrated a positive cause‐effect relationship between the amplitude of the circadian peak dopaminergic activity directly at the biological clock (suprachiasmatic nucleus; SCN) area and the level of glucose tolerance in animals^1^, ^3^, ^7^ [reviewed in ref. 8]. A diminution of the circadian peak of dopaminergic activity at the SCN facilitates a chronic increase in noradrenergic (and serotonergic) activity at the ventromedial hypothalamus (VMH) which in turn in and of itself is sufficient to induce the entire metabolic syndrome (obesity, insulin resistance, glucose intolerance, leptin resistance and hypertension) without alteration of caloric intake.^3^, ^6^, ^9^, ^10^ Notably, an increase in VMH norepinephrine level/action is a hallmark of the metabolic syndrome across a wide variety of animal models of the disorder [reviewed in ref. 8]. Both appropriately circadian‐timed dopamine administration directly into the SCN area and systemic (or intracerebroventricular) bromocriptine treatment of insulin‐resistant, glucose‐intolerant animals to reinstate the brain and SCN circadian peak of dopaminergic activity observed in healthy animals reverse (reduce) the elevated VMH norepinephrine and serotonin function profiles to normal and also ameliorate the glucose‐intolerant condition.^2^, ^3^, ^4^, ^6^, ^11^, ^12^ These findings suggest that the dopamine‐clock system somehow controls the brain's modulation of peripheral metabolism and its responsiveness to local glucose changes to effectuate such modulation of metabolism.

The VMH is a prominent glucose‐sensing centre^13^, ^14^, ^15^, ^16^, ^17^ that continually monitors the brain's current glucose fuel state, principally through two glucose‐sensing cell populations termed glucose inhibitory (GI) and glucose excitatory (GE) neurons. In normal healthy rats, brain (including VMH) glucose levels are about 30% of the peripheral glucose level and vary from about 0.7 mM to as high as 5.0 mM depending upon the degree and nature of fasting or feeding, respectively (14). An ambient rat VMH glucose concentration of as high as about 5 mM can be typical after a meal.^18^, ^19^ VMH GI neurons increase their electrical activity as the ambient VMH glucose level decreases below ambient euglycaemia (1.5‐2.5 mM in the brain) resulting in activation of neural circuits that stimulate an increase in sympathetic activity to the liver and adrenal medulla, as well as to pancreatic alpha cells to induce glucagon secretion, all to increase hepatic glucose output to counter the hypoglycaemia and re‐establish the euglycaemic state for the body and particularly for the brain (the classical ‘counter regulatory response’ [CCR]^13^, ^14^, ^16^). This local VMH hypoglycaemia‐induced CCR is mediated in large part by an increase in local noradrenergic activity at the VMH.^20^ Conversely, these VMH GI neurons decrease their electrical activity as the ambient glucose level increases towards and above euglycaemia.^13^, ^14^, ^16^ The VMH GE neurons of normal healthy animals increase their electrical activity as the VMH ambient glucose level rises towards and above euglycaemia which functions to facilitate neuroendocrine events that promote (insulin dependent and independent) postprandial glucose disposal in the periphery.^13^, ^14^, ^16^ As such, activation of VMH GE neurons plays a role in maintaining normal glucose tolerance. These GE neurons decrease their electrical activity as the ambient glucose levels decrease towards and below euglycaemia.^13^, ^14^, ^16^ The intracellular mechanisms involved in signal‐response coupling differ for GI and GE neurons, and though incompletely understood, have been reviewed extensively.^13^, ^14^, ^16^ In general, a reduction of ambient glucose at VMH GI neurons results in AMP‐activated protein kinase (AMPK) activation and resultant nitric oxide‐induced activation of soluble guanylate cyclase leading to chloride channel (cystic fibrosis transmembrane regulator) closure and depolarization and the subsequent CCR while a rise in ambient glucose level at VMH GI neurons normally results in inhibition of AMPK. At the VMH GE neuron, a rise in glucose appears to induce uncoupling protein 2 (UCP2) activity that is required for the subsequent increase in peripheral glucose disposal and insulin sensitivity.^21^ These data respecting delineated VMH glucose‐sensing responses of GE and GI neurons to local extracellular glucose fluxes have been derived primarily from in vitro experiments of isolated VMH brain slices or neurons.^13^, ^14^, ^16^ Certain of these studies suggest that in insulin‐resistant states, the VMH glucose‐sensing apparatus in vivo could be altered in a manner that may facilitate insulin resistance,^22^, ^23^ as previously suggested.^8^ However, neither the influence of a metabolic syndrome inducing (eg high fat) diet on the nature of the electrophysiological response of VMH GE and GI neurons to both increasing and decreasing alterations in the local VMH glucose concentration, respectively, nor the impact of circadian‐timed dopamine agonist administration on such VMH glucose‐sensing neuron responses among high‐fat diet (HFD)‐induced obese animals have ever been investigated in the intact animal in vivo. In this regard, however, sustained high‐fat feeding of rats for several weeks has been demonstrated to both reduce the circadian peak of SCN dopaminergic activity and increase VMH noradrenergic activity leading to the glucose‐intolerant condition.^3^ Therefore, given the cause‐effect relationship between sensitivity to the fattening effects of high‐fat diet feeding to diminish the circadian peak of dopaminergic activity at the SCN and consequently increase VMH noradrenergic activity to induce glucose intolerance, we hypothesized (a) that a sustained high‐fat diet would alter VMH GE and GI neuronal electrophysiological responses to physiological increases and decreases in ambient VMH glucose levels, respectively, in a manner that would potentiate insulin resistance and glucose intolerance (decrease VMH GE responsiveness to ambient glucose increase and increase VMH GI responsiveness to ambient glucose decrease) and (b) that appropriately circadian‐timed dopamine agonist (bromocriptine) treatment that mimicked the natural circadian peak of brain dopaminergic activity (but not such bromocriptine treatment outside this circadian window) would reverse the adverse effects of the high‐fat diet on VMH GE and GI neuronal glucose sensing and glucose intolerance even while the animals are maintained on the high‐fat diet. The current study tested these hypotheses via in vivo recordings of VMH GE and GI neurons firstly in rats maintained on normal versus obesogenic high‐fat diet and secondly in those animals on obesogenic high‐fat diet and treated with vehicle or such varying circadian‐timed bromocriptine administration, respectively. The influence of norepinephrine upon VMH GE neuronal responses to local hyperglycaemia in regular chow‐fed animals was also investigated.

## 2 MATERIALS AND METHODS

### 2.1 Animals

Female Sprague‐Dawley (SD) rats (Taconic Biosciences) (at 12‐16 weeks of age) were employed in all studies. Rats were singly housed in transparent cages and maintained on a 14‐hour daily photoperiod (14:10 light/dark [LD] cycle) for at last one week prior to initiation of experimentation and throughout the study period while allowed to feed and drink ad libitum. To avoid the confounder of age‐induced insulin resistance upon the background metabolic status of the study animals during the study time period, female rats were used inasmuch as they maintain a steady state of insulin sensitivity for a long period of their lifetime versus male rats of this strain that develop insulin resistance progressively from an early age.^24^, ^25^ Influence of oestrous cycle day on study outcomes was minimized by random daily investigation of random animals within the study groups over a 4‐month time period. It has previously been observed that oestrous cycle day does not influence VMH GE neuronal response to glucose.^26^ Similarly, animals were held on a 14‐hour daily photoperiods to avoid the confounder of short (12 hours) daily photoperiod‐induced insulin resistance upon the background metabolic status of the study animals during the study time period.^27^

All experiments were conducted according to policy reviewed by the VeroScience institutional animal care and use committee.

### 2.2 Study designs

#### 2.2.1 Study 1—Effect of HFD on VMH GE and GI glucose‐sensing neurons

Rats at 7 weeks of age were maintained on daily 14:10 light:dark cycles in a climate‐controlled facility while fed ad libitum either a high‐fat diet (HFD) (60% of calories from fat (90.7% lard, 9.3% soybean oil) per gram of food; 20% protein, 20% carbohydrates (Lodex10, fine granulated sucrose), 5.21 kcal/g, Research Diets Inc, catalog # D12492) or regular chow (RC) (18% of calories from fat (soybean oil) per gram of food; 24% protein, 58% carbohydrates (wheat, corn, soybean), 3.1 kcal/g, Teklad, Envigo WI catalog # 2018) for 5 weeks. After 5 weeks of feeding the HFD, animals were selected as HFD‐sensitive – diet induced obese by the criterion of a minimum 35% weight gain (N = 10), and used for in vivo electrophysiological recordings of VMH GE and GI neurons while maintained on the HFD until neuronal experimentation as described below. A parallel group of lean RC feed rats (N = 10) served as the comparator control group for such electrophysiological recordings.

#### 2.2.2 Study 2—Effect of circadian‐timed bromocriptine treatment of obese, insulin‐resistant HFD‐fed rats on VMH GE and GI glucose‐sensing neurons and glucose tolerance

Twelve‐week‐old animals made diet‐induced obese, insulin‐resistant by HFD feeding for the prior 5 weeks and maintained as in Study 1 was divided into four groups (n = 5‐8 per group) and treated at either Zietgiber Time (ZT; hours after light onset) 13.5 or ZT 8 with either bromocriptine (10 mg/kg body weight; in 30:70 EtOH:Sterile water solution; half‐life of approximately 90‐120 minutes) or vehicle given intraperitoneally for 14 days. After 14 days of such treatment in vivo, electrophysiological recordings from VMH glucose‐sensing neurons from animals of all four groups were conducted at ZT 14 on day 15 of the study as described below. A subsequent second group of animals similarly treated with bromocriptine or vehicle at ZT 13.5 were subjected to a glucose tolerance test on day 15 as follows. Electrophysiological recording and GTT experiments on day 15 occurred at least 24 hour following an animal’s last injection of either bromocriptine or vehicle. Electrophysiological recording and GTT experiments on day 15 occurred at least 24 hour following an animal’s last injection of either bromocriptine or vehicle. Rats (10‐11 weeks of age) were housed individually and maintained on a daily 14:10 LD cycle with HFD (d12079B, Research Diet Inc) and water provided ad libitum. The obese‐prone rats were selected based on body weight gain of minimum 35% after one month of feeding on HFD (body weight = 369 ± 7.9 g) and were randomly divided into 2 groups (Vehicle, N = 10; bromocriptine, N = 10) and received drug treatment daily at ZT 13.5 for 14 days. Bromocriptine mesylate was prepared in 20% ethanol/water solution and injected at 7.5 mg/kg (intraperitoneal, IP) while the vehicle rats received the same volume of 20% ethanol/water IP. Glucose tolerance test (GTT) was performed at the end of 14 days of treatment (as in the electrophysiological studies) during the fasting period of the day (6 hours after light onset and after a 6‐hour fast). On the day of GTT, the rats were fasted for 6 hours before receiving glucose injection (3 g/kg body weight, IP) and the tail blood was collected before and at 30, 60, 90 and 120 minutes after glucose injection for determination of blood glucose (One‐touch glucometer, LifeScan Inc, catalog # AW 06398902A) and plasma insulin (Elisa, Alpco catalog #80‐INSMR‐CH01). Body weight and food consumption were monitored throughout the experiment.

#### 2.2.3 In vivo VMH electrophysiological recordings from glucose‐sensing neurons for studies 1 and 2

##### Neuronal identification approach

Inasmuch as VMH GI and GE neurons are disbursed throughout the VMH with certain concentrations at the dorsomedial and ventrolateral regions, respectively,^13^, ^14^, ^16^ we placed the agent infusion cannula and recording electrode in the mediolateral region to sample both GI and GE neuronal activity. To isolate and identify VMH GE neuronal responses to ambient glucose increase and GI neuronal responses to ambient glucose decrease in vivo, we employed a modification of a previously established in vivo electrophysiological recording system from VMH neurons^19^ as follows. Since VMH GE neurons, but not GI neurons, exhibit an increase in electrical activity above baseline with an increase in VMH ambient glucose concentrations above euglycaemia (1.5‐2.5 mM awake and 2.5 mM anaesthetized corresponding to about a 4.5‐7.5 mM peripheral glucose concentration, respectively),^14^, ^28^, ^29^ extracellular multi‐unit recordings from platinum tungsten electrodes were made within the VMH during the micro‐application of step‐wise increasing glucose levels by application of 1 mM up to 5 mM glucose in the immediate vicinity of the electrode (within ~100 um based upon impedance) once a consistently maintained baseline level of activity was established. Similarly, since VMH GI neurons, but not GE neurons, exhibit an increase in electrical activity above baseline (including post‐ and presynaptic facilitated excitation) with a decrease in ambient glucose levels below euglycaemia (2.5 mM anaesthetized),^23^, ^29^ extracellular multi‐unit recordings from platinum tungsten electrodes were made within the VMH during the micro‐application of step‐wise increasing concentrations of 2‐deoxyglucose (2DG) from 1 mM up to 15 mM [thereby increasingly reducing ambient glucose concentration] in the immediate vicinity of the electrode (within ~100 µm). The local extracellular metabolizable glucose level available to the neuronal tissue estimated at 2.5 mM at baseline is diluted and displaced by the 2DG administered dose. As such, the local fractional useable glucose concentration following the 2DG administration is calculated as [baseline glucose] ([baseline glucose]/[baseline glucose plus 2DG]) × 0.5 (volume dilution factor) to become estimated between 0.9 and 0.175 mM in the presence of increasing concentrations of 2DG from 1 to 15 mM used in the study. Electrophysiological recordings within the VMH at 14 hours after light onset (ZT 14, the onset of darkness which is the natural daily feeding initiation time in these nocturnal animals) in response to increasing locally administered levels of glucose were made to simulate local physiological hyperglycaemia to test for responsiveness to local physiological postprandial hyperglycaemia. Similarly, such recordings in response to increasing local concentrations of 2‐deoxyglucose (2‐DG) were made to simulate local physiological hypoglycaemia to test for responsiveness of GI neurons that increase activity in response to local glucopenia^28^, ^29^ in the same test animals.

##### Animal surgery

On the day of electrophysiological recording, blood glucose levels were monitored (One‐touch glucometer, LifeScan Inc, catalog # AW 06398902A) and then animals were anaesthetized under 100 mg/kg thiobutabarbital (inactin) and mounted into a stereotaxic frame. Body temperature was maintained at 37°C throughout surgery and subsequent electrophysiological experimentation via animal placement upon a heating pad. A midline incision was made to expose the underlying skull. A small craniotomy ~1.5 mm in diameter was made over the left or right hemisphere, to allow access to the VMH (centred 2.8 mm posterior and 0.5 mm lateral of Bregma), and a second 1 mm crantiotomy was made 4‐5 mm posterior of Bregma and 2‐4 mm lateral of the midline in the contralateral hemisphere where a cranial screw was placed to serve as ground/reference. A recovery period from surgery of 20 minutes was allowed after the initiation of electrode placement and the beginning of neuronal electrical recordings. Placement of the cannula and recording electrode was verified by electrolytic induction of microlesions at the VMH by passing 150 µA DC for 60 seconds through recording electrodes. Frozen sections were stained with haematoxylin and compared to a rat brain atlas for verification.^30^ Following the termination of electrophysiological recordings, animals were sacrificed by anaesthesia overdose.

##### Electrophysiologic recording and data acquisition

Recording electrodes were constructed from 80 µm quartz‐insulated platinum tungsten (Thomas Recording, catalog #AN000123) with a 25 µm core and were sharpened to a fine tip on a rotating (60‐100 Hz) diamond abrasive plate (Sutter Instruments, catalog #104D), sharpened with a length of ~250 µm. Methods for construction of this ‘Reitböck’ style electrode have been described elsewhere^31^, ^32^ with the exception that the present electrodes were left un‐pulled to generate larger tips of lower impedance (impedance, 0.3‐0.8 Mohm) better suited for multi‐unit recording from small populations of neurons.

A double‐barreled guide stainless steel cannula was fabricated by attaching a 27 gauge stainless steel hyperdermic tube (27 gauge) to a PlasticsOne external gauge cannula (24 gauge) to aid in the insertion of electrode, and the drug delivery cannula, respectively. A miniature single‐channel microdrive^33^ was attached to the cannula system to allow independent movement of the electrode with respect to the infusion cannula and to allow the electrode to be slowly lowered into the VMH (depth of 9.4 mm from the Dura, −2.8 AP, 0.5 ML) just prior to initiation of recordings.

Once the guide system was placed, it was anchored to the animal using a dental cement bridge to the ground screw. With the cement hardened, the internal drug delivery cannula (31 gauge, 2 mm longer than the external cannula) was prefilled with the compound to be delivered (either glucose or 2‐DG) and inserted into the external guide cannula. The electrode was lowered 2 mm beyond the guide cannula via the attached microdrive, and the depth adjusted (~100 µm) until a dense multi‐unit activity was present in the recording.

Following the lowering of the electrode to the appropriate placement within the VMH, the electrode was left in place for 20 minutes for neural activity to stabilize. A baseline measure of spontaneous multi‐unit activity was then recorded for at least 60 seconds. Baseline measures were compared with those obtained during 1 µL injections of artificial cerebral spinal fluid containing 1‐5 mM (1‐5 nmol/μL) glucose or 1‐15 mM (1‐15 nmol/μL) 2DG that were delivered over a one minute time period.

A rest period of approximately 15 minutes separated the injection periods of glucose to allow recovery to baseline firing rates during which time the internal cannula was replaced with another prefilled internal cannula with a higher concentration of glucose. Then after the glucose dose challenge was complete and an additional rest period, 2‐DG was similarly administered in increasing concentrations with the same rest and injection time period sequence.

Electrical signals were passed through an amplifier, surveyed through an AD instrument powerlab with a NeuroEx signal conditional (AD instruments, CO, catalog # FE185). Electrical signals were filtered at 0.3 Hz – 5 kHz and sampled at 10 or 20 kHz. Spike detection was accomplished using a threshold discriminator and then cluster cutting of selected electrophysiologic signals was conducted offline with the use of Lab Chart 8 (CO, catalog # MLU260M/8) to isolate spike potentials from the background data. Multi‐unit activity was quantified as events per second for all selected spike potentials. Responses to glucose or 2‐DG were measured as the per cent change in firing rate during the injection period relative to the pre‐injection baseline.

#### 2.2.4 Study 3—Effect of local VMH norepinephrine application on the responsiveness of VMH GE neurons to local glucose increase

##### Animal surgery

Female Sprague‐Dawley rats at 10‐12 weeks of age (N = 10) were employed in this study. Rats were singly housed in transparent cages and maintained on a 14‐hour daily photoperiod (14:10 light/dark cycle) for 2 weeks prior to initiation of experimentation and throughout the study period while allowed to feed RC and drink water ad libitum. On the day of recording, blood glucose levels were monitored (One‐touch glucometer, LifeScan Inc, Switzerland catalog # AW 06398902A), animals were anaesthetized under 100 mg/kg thiobutabarbital (inactin) and mounted into a stereotaxic frame. Body temperature was maintained at 37°C throughout experimentation via a heating pad. A midline incision was made to expose the underlying skull. A small craniotomy ~1.5 mm in diameter was made over the left or right hemisphere, to allow access to the ventromedial hypothalamic nucleus (centred 2.8 mm posterior and 0.5 mm lateral of Bregma), and a second 1 mm craniotomy was made 4‐5 mm posterior of Bregma and 2‐4 mm lateral of the midline in the contralateral hemisphere where a cranial screw was placed to serve as ground/reference. Dura was removed, a three‐barrel micro‐pipette with central barrel filled with a low‐impedance carbon fibre recording electrode (Carbostar, Kation Scientific) was implanted into the VMH was lowered to 9.4 mm ventral to Bregma.

##### Electrophysiologic recording and data acquisition

Separate drug administration pipettes were prefilled with de‐ionized water containing either 0.5 M glucose or 0.5 M norepinephrine and given a holding current of +10nA. The iontophoretic injection current for glucose employed to elicit MUA from VMH GE neurons was at 100 nA (determined from initial dose‐response testing). The minimum iontophoretic injection current for norepinephrine to elicit an effect on VMH GE response to this glucose administration was at 40 nA. Extracellular voltages were recorded for five‐minute‐long recording sessions during which time glucose with or without norepinephrine was iontophoretically applied for 60‐second injection periods. After recording, action potentials were identified with a threshold discriminator, and then subsequently spike sorted using cluster analysis. Electrical signals were passed through an amplifier, surveyed through an AD instruments powerlab with NeuroEx signal conditional (AD instruments, Colorado, catalog # FE185). Electrical signals were filtered at 0.3 Hz – 5 kHz, and sampled at 20 kHz. Spike detection was accomplished using a threshold discriminator and then cluster cutting of selected electrophysiologic signals was conducted offline with the use of Lab Chart 8 (AD instruments, Colorado, catalog # MLU260M/8) to isolate spike potentials from the background data. Multi‐unit activity was quantified as events per second for all selected spike potentials. Responses to glucose were measured as per cent change in firing rate during the injection period relative to the preinjection baseline.

Spontaneous activity was recorded for 60 seconds to establish basal levels of activity.

Following baseline measures, glucose was iontophoretically applied at 100 nA for 60 seconds to measure the responsiveness of GE neurons to increases in local glucose. A 60‐second rest period was next given to allow the neurons to return to basal firing rates, and then norepinephrine was iontophoretically applied for the next 2 minutes via the second injection barrel (40 nA). During the last minute of NE application, glucose was applied for a second time to evaluate the responsiveness of GE neurons during local NE application. For each session, the multi‐unit firing rate (MUR) was determined by applying a threshold discriminator to identify action potentials, and counting action potentials for each 1 second bin in the recording time period.

### 2.3 Statistical analyses

Parametric tests were used only when groups passed normality (using the Shapiro‐Wilk test). Dose‐response curves were first analysed by ANOVA for a main effect, and post hoc testing at independent dose levels was conducted using a Bonferroni correction. In all figures and text, data are presented as mean ± SE for recorded data from animals per treatment group or for responses within animals after vs. before a particular treatment administration, with p values. A significant difference between test groups was accepted at the *P* < .05 level.

## 3 RESULTS

### 3.1 Study 1**—**Effect of HFD on VMH GE and GI glucose‐sensing neurons

**FIGURE 1:**
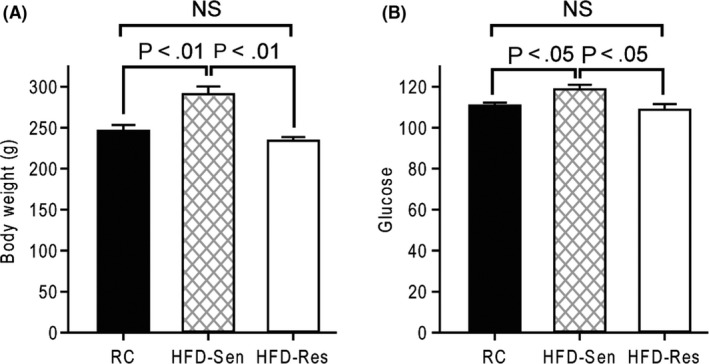
Rats sensitive to the obesogenic effects of HFD feeding (HFD‐Sen) increase body weight and blood glucose relative to HFD‐resistant (HFD‐Res) rats and regular chow (RC) fed rats. Body weight (A) and blood glucose (B) were measured among rats maintained on RC (black bar) or high‐fat diet for five weeks. Animals on high‐fat diet were separated by weight into high‐fat diet‐sensitive (hatched bar) and high‐fat diet‐resistant (white bar) by criteria described in methods

Studies were conducted to determine the effect of an obesogenic high‐fat diet (versus regular chow diet) upon VMH GE and GI neuronal electrophysiological response and sensitivity to local hyperglycaemia and hypoglycaemia, respectively. Rats selected as high‐fat diet‐sensitive – diet induced obese were significantly heavier (17.6%, from 246.3 to 291.0 g, *P* < .01, Figure 1A) and had blood glucose levels significantly higher (7.2%, from 110.8 to 118.8 mg/dL, *P* < .05, Figure 1B) than rats maintained on regular chow at the time of VMH neuronal recordings.

**FIGURE 2:**
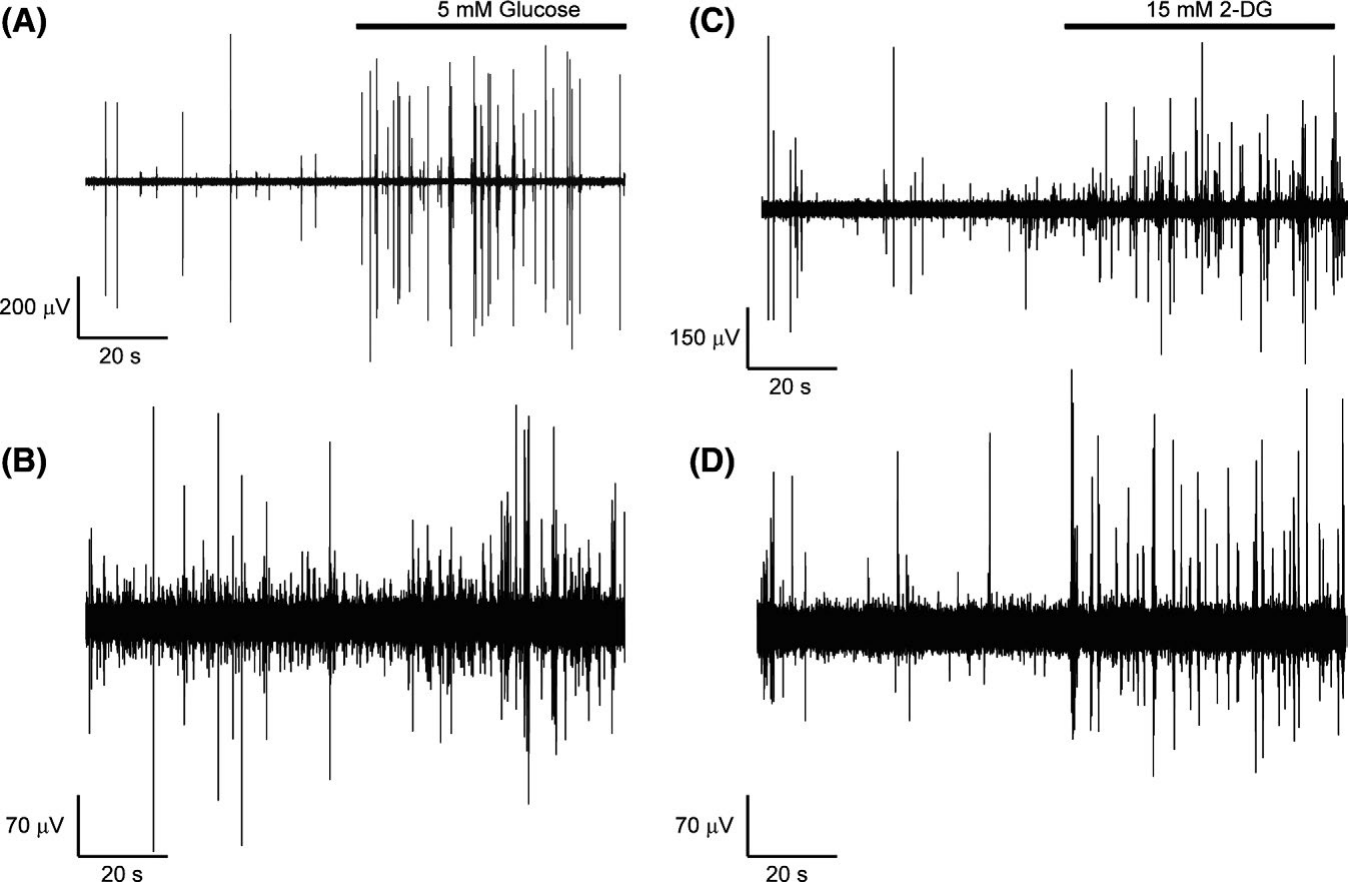
Exemplary recordings of 5 mM (A, B) glucose stimulated or 15 mM 2‐DG (C,D) stimulated VMH neuronal activity over a one minute recording period obtained from a rat maintained on a regular chow diet (A, C respectively) or obesogenic HFD (B, D respectively). Response to 5 mM glucose (A and B) or 15 mM 2‐DG (C and D) infused near the recording electrode indicated by the horizontal bar

Figure 2A,B depict exemplary VMH neuronal multi‐unit recordings for baseline and glucose‐stimulated responses to ambient 5 mM glucose from a rat fed RC (A) or obesogenic HFD (B) for 5 weeks. Note a greater increase in number of multi‐unit events per unit time (1 minute) in response to local 5 mM glucose infusion obtained from a rat maintained on a RC diet (Figure 2A), versus such events from a recording obtained from a rat maintained on an obesogenic HFD (Figure 2B). Figure 2C,D depict exemplary VMH neuronal multi‐unit recordings for baseline, and 2‐DG stimulated responses to ambient 15 mM 2‐DG from a rat fed RC (C) or obesogenic HFD (D) for 5 weeks. Note a greater increase in number of multi‐unit events per unit time (1 minute) in response to local 15 mM 2‐DG infusion obtained from a rat maintained on an obesogenic HFD (Figure 2D), versus such events from a recording obtained from a rat maintained on RC (Figure 2C).

**FIGURE 3:**
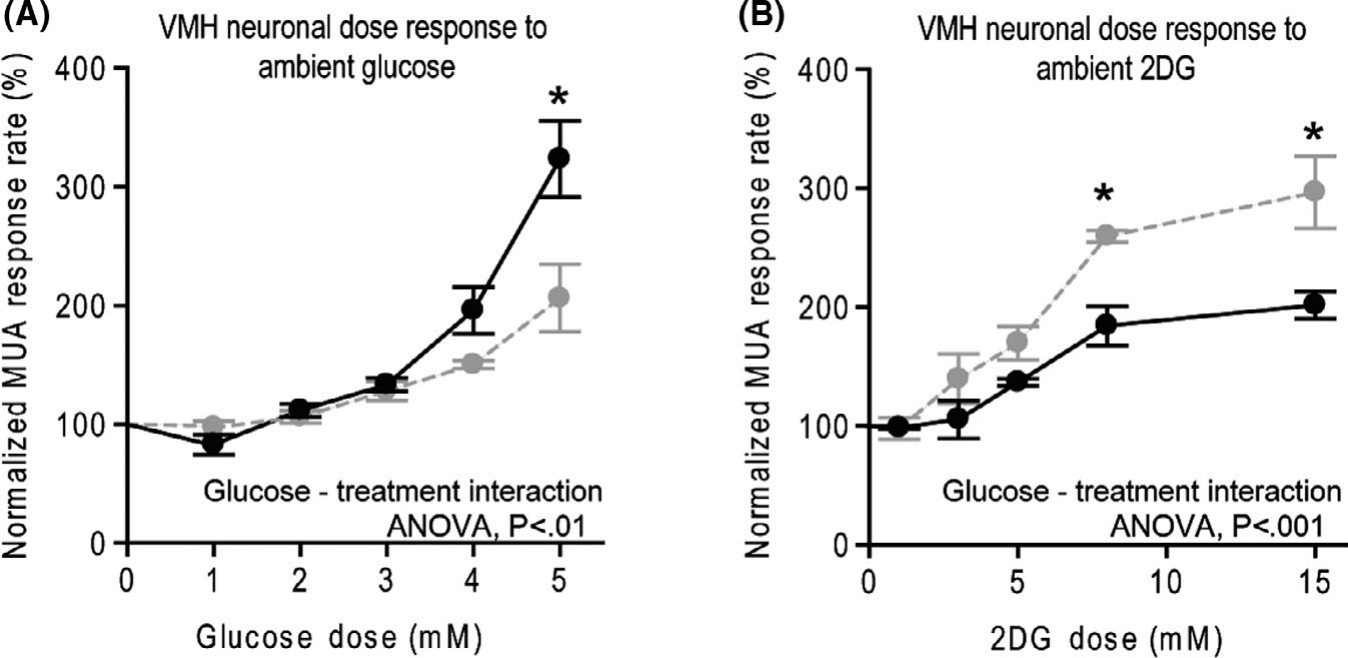
Glucose sensing of VMH neurons in vivo is altered by a history of obesogenic high‐fat diet. A, A normalized electrophysiological (multi‐unit activity, MUA) response (proportional to baseline) of VMH neurons to varying doses of locally infused glucose from rats maintained on a regular chow diet (solid line) or on a high‐fat diet (dashed line) for five weeks. B, Normalized electrophysiological (activity, MUA) response (proportional to baseline) of VMH neurons to varying doses of locally infused 2‐DG from rats maintained on a regular chow diet (solid line) or on a high‐fat diet (dashed line). An asterisk denotes a significant difference from RC control at *P* < .05, post hoc with bonferroni correction. See results section for full statistical analysis description

The complete ambient glucose dose ‐ VMH GE neuron electrophysiological response curve for obese HFD sensitive versus RC diet animals is presented in Figure 3A. There was a significant main effect of glucose dose (ANOVA, *F* = 33.08, *P *< .001), and a significant main affect was seen such that multi‐unit responses to glucose across the dose‐response were higher for animals on regular chow versus on the high‐fat diet (ANOVA, *F* = 7.74, *P* < .05). There was a significant interaction between glucose dose‐response and treatment group (ANOVA, *F* = 4.71, *P* < .01). Post hoc analysis (Bonferroni) revealed these curves significantly departed at the 5 mM dose (*P* < .001). The stimulated multi‐unit response to 5 mM glucose was significantly reduced in rats maintained on a high‐fat diet versus rats on regular chow (Figure 3A, RC = 323.2 ± 32.1% increase, HFD = 206.0 ± 28.5% increase Student's *t*, *P* < .05).

The 2‐DG dose – VMH GI neuron electrophysiological response curve for obese HFD sensitive versus RC diet animals is presented in Figure 3B. There was a significant main effect for drug dose (*F* = 31.36, *P* < .001) and a significant main effect for diet such that multi‐unit responses to 2‐DG across the dose‐response were greater for animals on the obesogenic high‐fat diet versus regular chow diet (ANOVA, *F* = 19.63, *P* < .001). Post hoc analysis (Bonferroni) revealed these curves significantly departed at both the 7.5 mM (*P* < .01) and 15 mM doses (*P* < .001). The stimulated multi‐unit response to 15 mM 2‐DG was significantly greater in rats maintained on a high‐fat versus regular chow diet (Figure 3B, RC = 201.5 ± 11.6% increase, HFD = 296.4 ± 30.5% increase, Student's *t*, *P* < .05). There was no between‐group difference in baseline activity prior to the glucose or 2‐DG infusions.

Receiver operating characteristic (ROC) analysis was performed on the MUA dose response curve data (both glucose and 2‐DG) to determine if the observed changes in responsiveness of GE and GI neurons (respectively) induced by HFD could translate into changes in signal detectability by downstream neurons. ROC analysis reaffirmed the general results from MUA dose response curves to both increasing local glucose and 2‐DG described above suggesting that such changes in MUA responsiveness of VMH GE and GI neurons, respectively, were sufficient enough to provide signalling detectable to downstream neurons that these VMH neurons communicate with (Supplemental Material).

In summary, exposure of rats to an obesogenic HFD for 5 weeks resulted in a simultaneous decrease in VMH GE neuronal sensitivity to glucose at physiological local VMH hyperglycaemia levels (ie observed after a meal) and an increase in VMH GI neuronal sensitivity to local hypoglycaemia. These concurrent events potentiate peripheral hyperglycaemia and insulin resistance (see Discussion for details).

### 3.2 Study2—Effect of circadian‐timed bromocriptine treatment of obese, insulin‐resistant HFD‐fed rats on VMH GE and GI glucose‐sensing neurons and glucose tolerance

**FIGURE 4:**
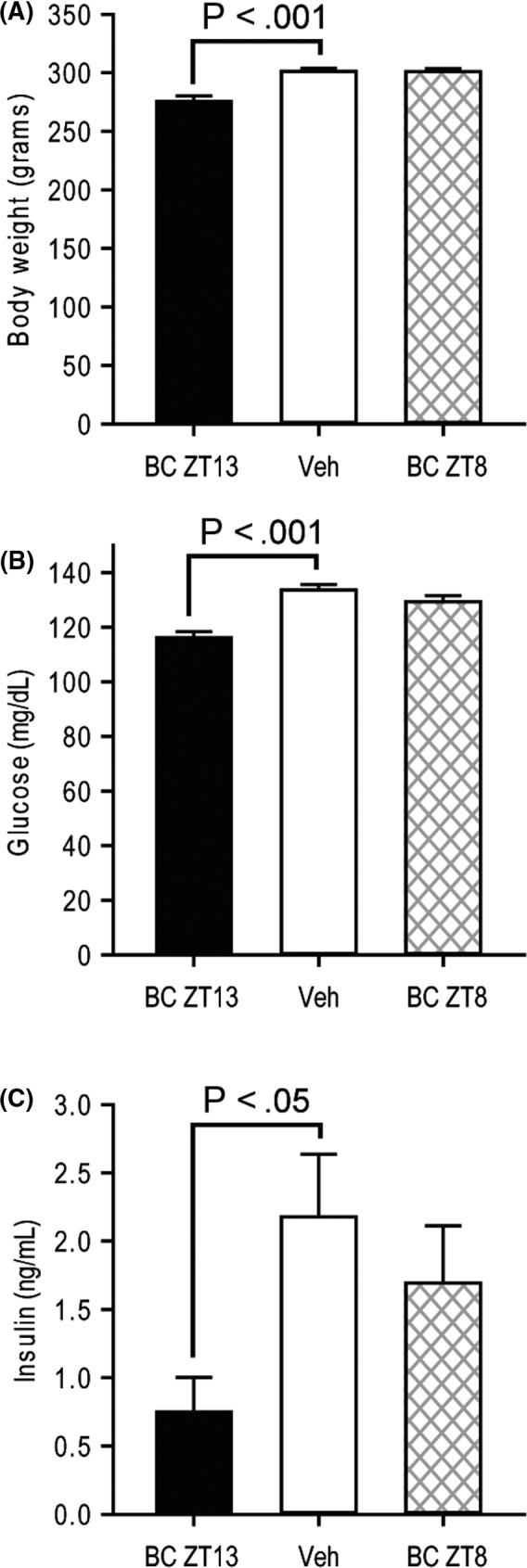
Circadian‐timed daily administration of bromocriptine reduces weight gain, blood glucose and plasma insulin levels of rats on an obesogenic HFD. (A) Body weight, (B) blood glucose or (C) plasma insulin of rats following five weeks of high‐fat diet, and subsequent 2 wk receiving bromocriptine at ZT13 (BC ZT13, black bar), vehicle (Veh, white bar) or bromocriptine at ZT8 (BC ZT 8, hatched bar)

**FIGURE 5:**
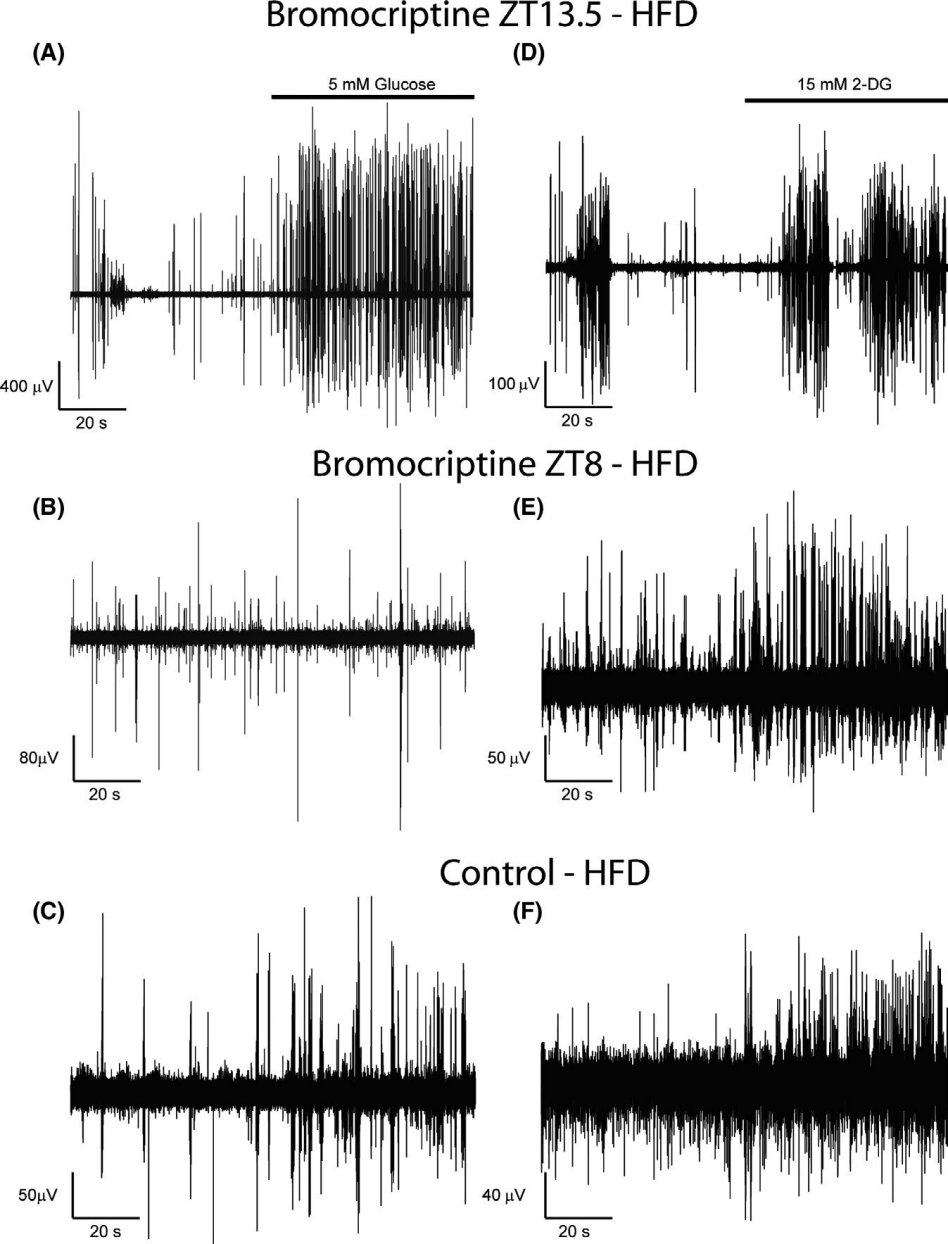
The obesogenic high‐fat diet‐induced reduction in VMH GE ambient glucose sensing is reversed by appropriate circadian‐timed bromocriptine administration. Example multi‐unit activity of VMH GE neurons in response to local 5 mM glucose infusion over a minute recording period obtained from either a rat maintained on a high‐fat diet that received daily injection of bromocriptine at ZT13.5 (A, just prior to the onset of locomotor activity), or such rat that received bromocriptine at ZT8 (B, the middle of rats sleep cycle) or such rat that received vehicle treatment (C). Example multi‐unit activity of VMH GI neurons in response to local 15mM 2‐DG infusion over a minute recording period obtained from either a rat maintained on a high‐fat diet that received daily injection of bromocriptine at ZT13.5 (D) or such rat that received bromocriptine at ZT8 (E) or such rat that received vehicle treatment (F). NOTE: data are analysed and expressed as the between group difference in changes from baseline MUA

Studies were conducted to investigate the impact of circadian‐timed (either ZT 8 or 13.5) bromocriptine administration to ameliorate the high‐fat diet‐induced diabetogenic alterations in VMH GE and GI neuronal responsiveness to local hyperglycaemia and hypoglycaemia, respectively. Bromocriptine treatment at ZT 13.5 (to mimic the natural circadian peak in brain dopaminergic activity) but not at ZT 8 (outside this circadian dopaminergic activity peak window) reduced body weight (8.5%), blood glucose level (13.0%) and plasma insulin level (65.6%) relative to control rats while maintained on a HFD (*P* < .001, 0.001, and 0.05, respectively) (Figure 4). Exemplary VMH neuronal multi‐unit responses to ambient 5 mM glucose infusion for HFD‐fed animals receiving bromocriptine injections at ZT 13.5 and ZT 8 relative to controls are presented in Figure 5A‐C, respectively. Note the effect of bromocriptine at ZT 13.5 but not bromocriptine at ZT 8 to increase baseline multi‐unit activity versus control. Exemplary VMH neuronal multi‐unit responses to ambient 15 mM 2‐DG infusion for HFD‐fed animals receiving bromocriptine injections at ZT 13.5 and ZT 8 relative to controls are presented in Figure 5D‐F, respectively. Note the effect of bromocriptine at ZT 13.5 but not bromocriptine at ZT 8 to decrease baseline multi‐unit activity versus control.

**FIGURE 6:**
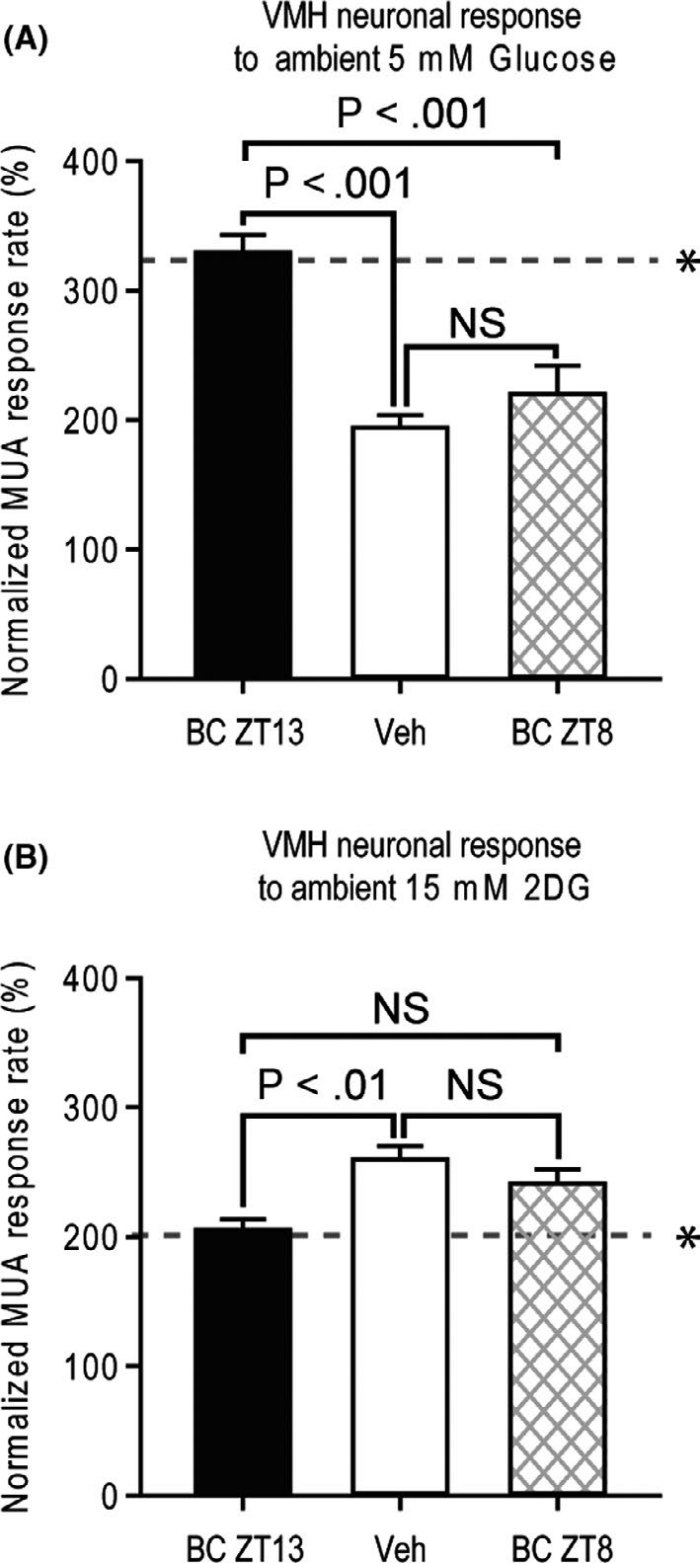
High‐fat diet‐induced changes in glucose sensing of VMH neurons (see Figure 3) are reversed by timed daily IP administration of bromocriptine. A, Normalized electrophysiological (multi‐unit activity, MUA) response (proportional to baseline) of VMH neurons to local injection of 5mM glucose from rats maintained on a high‐fat diet receiving bromocriptine at ZT13.5 (black bar), vehicle (white bar) or bromocriptine at ZT8 (hatched bar). B, Normalized electrophysiological (multi‐unit activity, MUA) response (proportional to baseline) of VMH neurons to local injection of 15 mM 2DG from rats maintained on a high‐fat diet receiving bromocriptine at ZT13 (back bar), vehicle (white bar) or bromocriptine at ZT8 (hatched bar). * dotted line depicts response in normal RC‐fed rats

The VMH GE neuronal electrophysiological activity response to ambient 5 mM glucose of obese HFD‐sensitive animals treated with either bromocriptine at ZT 13.5, bromocriptine at ZT 8 or vehicle is presented in Figure 6A. There was a significant main effect of treatment condition on the multi‐unit response to glucose infusion (ANOVA, *F* = 25.536, *P* < .001). Post hoc analysis (bonferroni) revealed that animals receiving bromocriptine injections at ZT 13.5 had a greater response to local infusion of 5 mM glucose than either rats receiving vehicle injections (by 171%, *P* < .001) or those receiving bromocriptine at ZT 8 (by 150%, *P* < .05). Bromocriptine administration at ZT 8 had no effect on the VMH neuronal response to glucose relative to vehicle control.

The VMH GI neuron electrophysiological response to ambient 15 mM 2‐DG of obese HFD‐sensitive animals treated with either bromocriptine at ZT 13.5, bromocriptine ZT 8 or vehicle is presented in Figure 6B. There was a significant main effect of treatment condition (ANOVA, *F* = 7.603, *P* < .01). Post hoc analysis (bonferroni) revealed that animals receiving bromocriptine injections at ZT 13.5 had a reduced response to local infusion of 15 mM 2‐DG than rats receiving vehicle (by 20%, *P* < .01). Bromocriptine administration at ZT 8 had no effect on the VMH neuronal response to 2‐DG relative to vehicle control.

**FIGURE 7:**
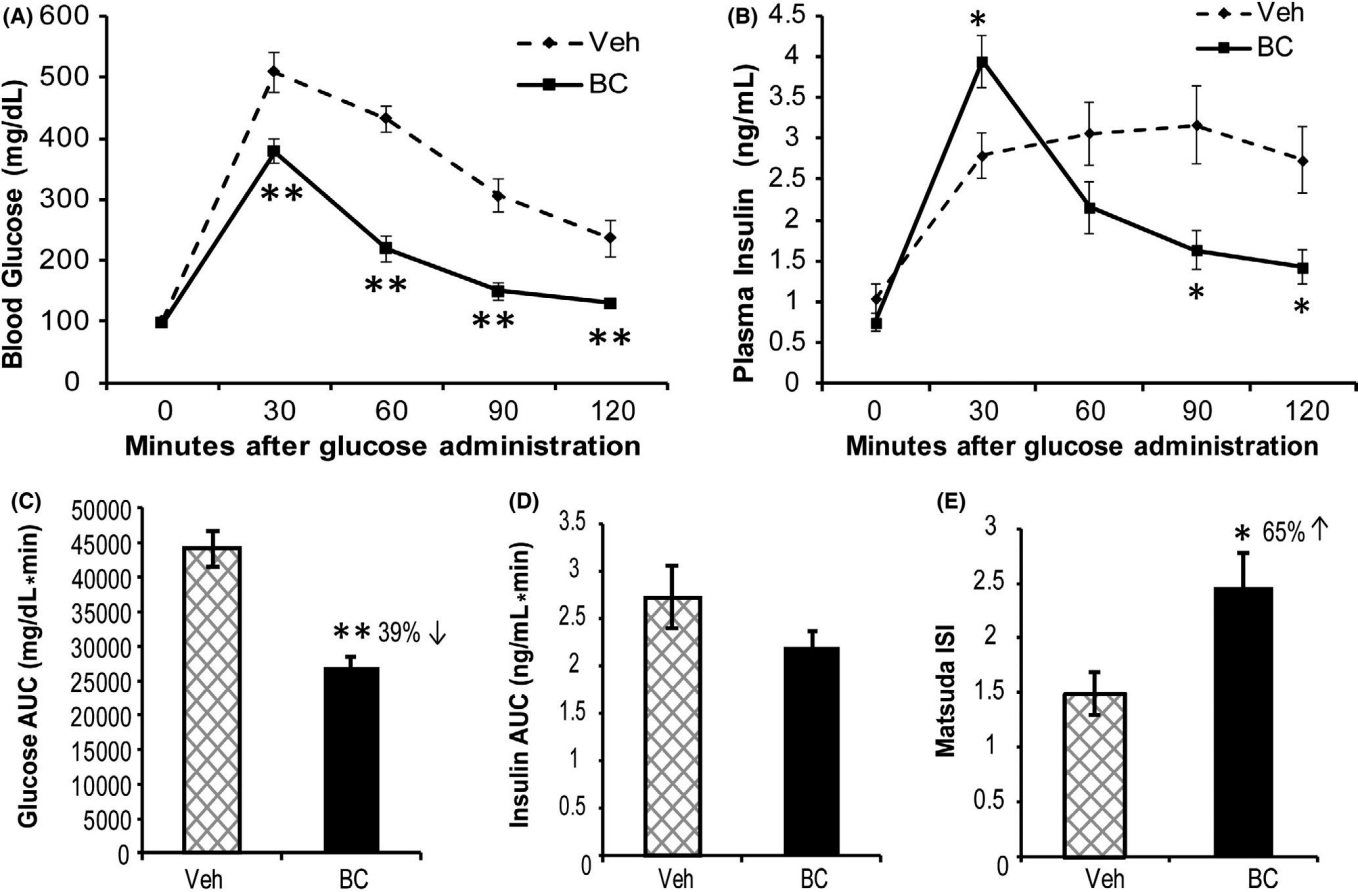
Bromocriptine treatment improves glucose tolerance and insulin sensitivity in HFD‐fed rats. A GTT was performed after 14 days of treatment with bromocriptine (BC) (7.5 mg/kg, ip) or vehicle (Veh) daily at the onset of locomotor activity (ZT13.5). Compared with vehicle, such bromocriptine treatment reduced blood glucose levels during the GTT (A) with a 39% reduction in glucose area under curve (AUC) (*P* < .0001) (C). Bromocriptine increased the GTT plasma insulin peak at 30 min while reducing plasma insulin levels at 90 and 120 min during the GTT (B) with insulin AUC reduced 19% (ns) (D). Bromocriptine increased insulin sensitivity by 65% as revealed by Matsuda ISI (*P* = .015) (E). An asterisk denotes a significant difference verses control (*P < .05, **P < .01)

The effect of bromocriptine on glucose homeostasis was assessed by GTT after 14 days of treatment. Compared with vehicle, bromocriptine improved glucose tolerance as revealed by a 39% reduction (*P* < .0001) in glucose area under curve (AUC) during the GTT. Although insulin AUC was not significantly reduced (*P* = .21), bromocriptine increased the plasma insulin peak level at 30 min (*P* = .015) and reduced plasma insulin levels at 90 mins (*P* = .018) and 120 mins (*P* = .018) after glucose challenge. As a result, bromocriptine increased Matsuda insulin sensitivity index by 65% (*P* = .00035) (Figure 7).

In summary, in rats maintained on an obesogenic HFD, daily bromocriptine treatment for 2 weeks timed to the natural circadian peak of endogenous dopamine activity in the brain of lean, insulin‐sensitive animals (ZT 13.5) that is diminished in obese, insulin‐resistant animals reversed the defects in both VMH GE and GI neuronal glucose sensing induced by the obesogenic diet (to increase GE hyposensitivity to glucose and decrease GI hypersensitivity to glucose both to normal levels). This effect of bromocriptine was completely absent when injected daily during the circadian trough of dopaminergic activity in the brain (ZT 8). The effect of bromocriptine treatment at ZT 13.5 on VMH glucose‐sensing neurons would be expected to be and was associated with an improvement in glucose tolerance and insulin sensitivity.

### 3.3 Study 3—Effect of local VMH norepinephrine administration upon VMH GE neurons local glucose sensing

**FIGURE 8:**
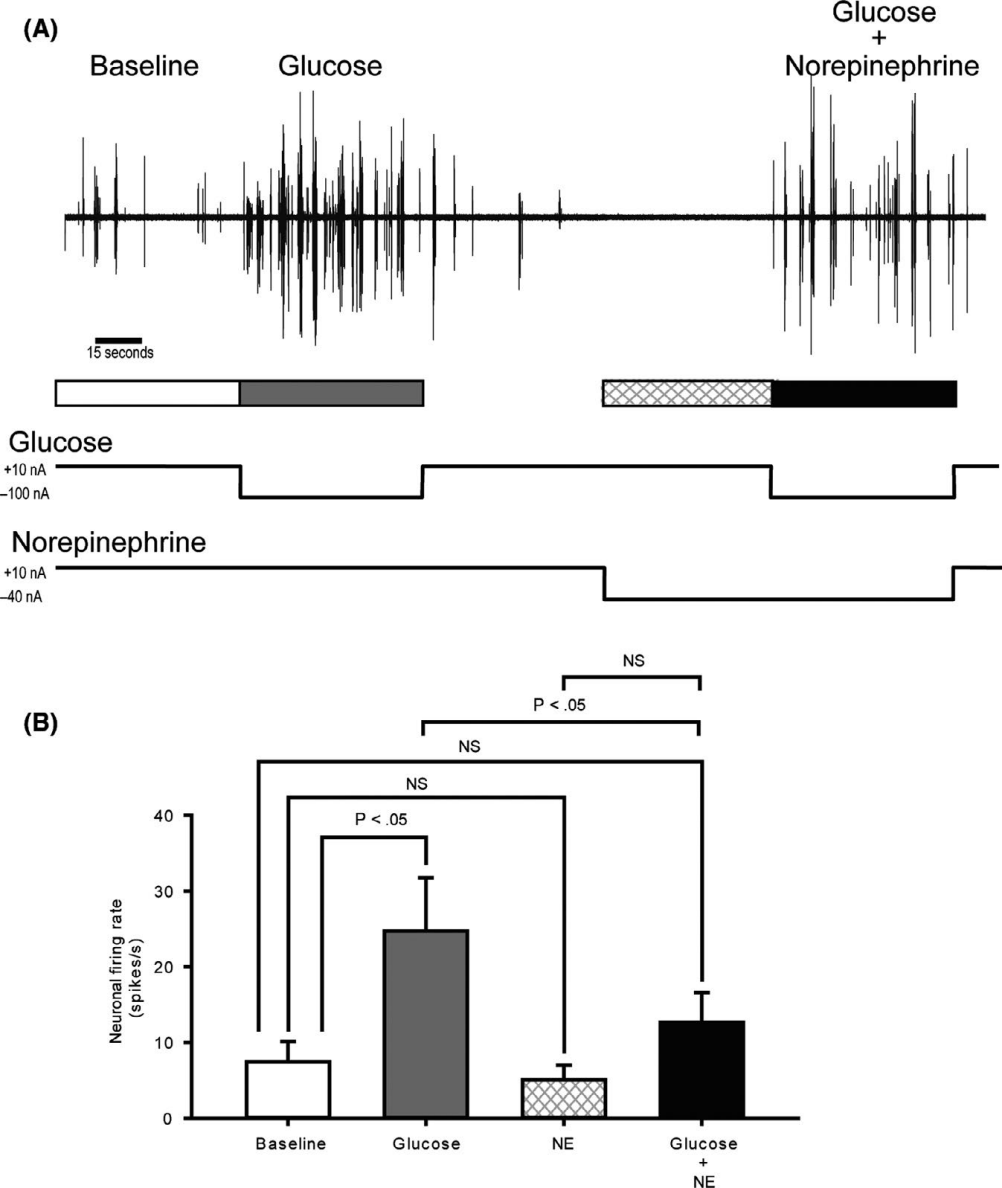
VMH GE neuronal response to local glucose increase is suppressed by local norepinephrine administration. A, Example voltage traces from five‐minute multi‐unit recordings (MUA) obtained from VMH. After 60 seconds of recording spontaneous activity (baseline firing rate, white), glucose was iontophoretically (electro‐osmotically) applied for 60 seconds (grey). After glucose injection, 60 seconds was given to allow cells to return to baseline firing rates (white), and then norepinephrine (NE) was iontophoretically applied initially for 60 seconds (hatched) and then continued for another 60 seconds during which time glucose was co‐administered (black). B, The average MUR from the VMH of 10 animals during baseline (white), glucose stimulation (grey), norepinephrine (hatched) and glucose plus norepinephrine pretreatment (black) conditions at the VMH. Error bars depict the s.e.m. See Methods section for details

Studies were conducted in regular chow‐fed animals to determine if VMH norepinephrine increase (typically observed in HFD‐fed animals and reduced by bromocriptine treatment) may abrogate the responsiveness of VMH GE neurons to local glucose increase as HFD feeding does (ie could increases in VMH norepinephrine observed with HFD feeding mediate the HFD effect to reduce VMH GE sensing of local hyperglycaemia and might bromocriptine ameliorate this HFD effect by reducing VMH norepinephrine). Iontophoretic application of glucose to the VMH increased baseline multi‐unit activity by approximately threefold (from 7.4 to 24.7 spikes/s; *P* < .05). After subsequent return to baseline, application of norepinephrine did not elicit any significant change in the multi‐unit activity from baseline. However, application of norepinephrine did reduce by 48.6% (*P* < .05) the absolute response of VMH GE neurons to subsequent local glucose infusion relative to such glucose infusion without pretreatment with norepinephrine (Figure 8).

The present findings demonstrate that increased noradrenergic activation at the VMH blocks VMH GE neuronal glucose sensing in animals on regular chow diet that resultantly would potentiate glucose intolerance. Since bromocriptine treatment has previously been shown to normalize elevated VMH noradrenergic activity in insulin‐resistant, glucose‐intolerant animals, the present study findings in composite suggest that this may be a mechanism by which bromocriptine improves VMH GE glucose sensing(and resultantly improves peripheral glucose tolerance) (see Discussion for details).

#### 3.3.1 Cannula and electrode placement analysis

**FIGURE 9:**
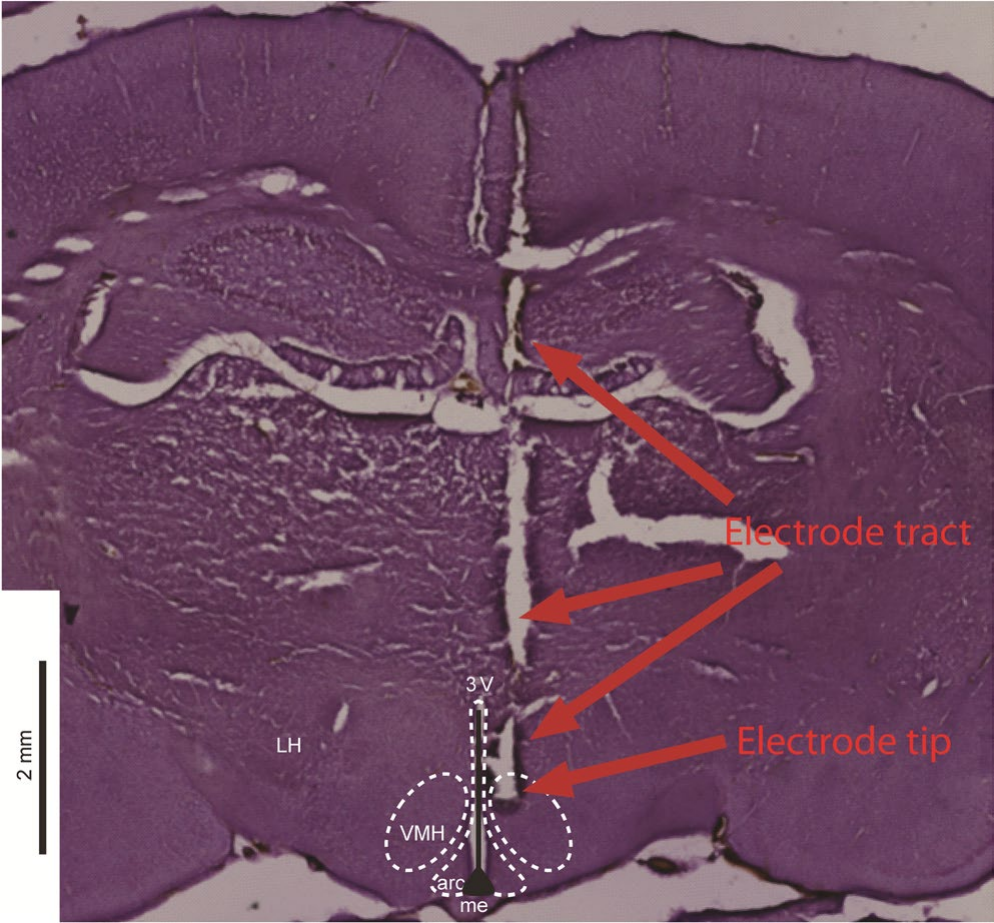
Electrode placement within the VMH. Electrode placement was verified via electrolytically induced microlesion at the electrode tip. Upper arrows indicate the tract of the electrode through dorsal tissue, and the lower arrow shows the region of the microlesion. Borders of relevant local brain regions are adapted from Paxinos and Watson,^30^ 3V = third ventricle, Arc = arcuate hypothalamic nucleus, LH = lateral hypothalamic area, ME = median eminence, VMH = ventromedial hypothalamic nucleus

Small (~200 µm) microlesions were induced at the tips of the recording electrodes by passing 150 µA DC, subsequently the brains were sectioned, and slices stained with haematoxylin to evaluate the placement of the recording electrode. An example microlesion induced at the recording area of VMH can be seen in Figure 9.

## 4 DISCUSSION

The present study is the first to demonstrate in vivo that (a) an insulin resistance/obesity‐inducing high‐fat diet simultaneously decreases the VMH GE neuronal excitatory responsiveness to local glucose infusion that approximates physiological postprandial increases in local VMH glucose levels (from 2.5 up to 5.0 mM)^18^, ^19^ and increases the VMH GI neuronal responsiveness to physiological decreases in local VMH glucose levels (to <2.5 mM) in a manner that would support a hyperglycaemic, glucose‐intolerant condition (Figures 2 and 3, see also Appendix S1) and (b) this neuropathophysiology can be substantially reversed by circadian‐timed dopamine agonist administration even in the sustained presence of the HFD. It should be noted that the in vivo dose‐responses of both local hyperglycaemia‐induced VMH GE neuronal activation and hypoglycaemia‐induced VMH GI neuronal activation among RC‐fed rats observed herein are in very good agreement with those previously reported in the pioneering and elegant work of Silver and Erecinska utilizing a different in vivo local glucose administration method and neuronal electrical recording technique.^19^

The blunted VMH GE excitatory responsiveness to local VMH hyperglycaemia among HFD‐fed obese vs. RC lean rats began to manifest at VMH glucose administration concentrations >3.0 mM and was clearly evident at 5 mM, which range equates to a normal brain postprandial glucose level or to plasma glucose during a standard 2 g/kg glucose tolerance test (GTT) in such normal RC‐fed rats (~9‐12 mM).^19^ Importantly, several studies implicate (glucose) activation of these VMH GE neurons to induce increases in peripheral insulin action and glucose disposal (reviewed in ref. 16). The observed diminution in VMH GE electrophysiological response to local physiological (postmeal) hyperglycaemia by HFD feeding would be expected to reduce the VMH GE neuronal activated induction of peripheral insulin action and glucose disposal following a meal.^16^ This observed diminution of VMH GE electrophysiological response to local hyperglycaemia is consistent with previous observations of decreased VMH Fos‐like immunoreactivity and glucose transporter 2 expression in response to peripheral hyperglycaemia among high‐fat diet‐induced obese insulin‐resistant rats, respectively.^34^, ^35^ Moreover, the present findings are also consistent with observations reported by Cotero et al^23^ of an *increased sensitivity* of cultured VMH GE neurons isolated from insulin‐resistant versus insulin‐sensitive rats *to the inhibitory effects of local hypoglycaemia* on their activity, an alteration that would potentiate a CRR at euglycaemic levels facilitating hyperglycaemia and glucose intolerance (ie when insulin resistance is present, GE neurons stop firing earlier as glucose levels decline in the postabsorptive state).

Congruent with the hyperglycaemia‐potentiating effect of obesity‐inducing HFD to inhibit VMH GE neuronal response to local hyperglycaemia, such dietary response was also coupled to increased VMH GI neuronal activation in response to physiological decreases in local glucose level (<2.5 mM) (versus response in RC rats) that if inappropriately activated at euglycaemia would also function to enhance peripheral hyperglycaemia via induction of the counter regulatory response mechanisms, as previously proposed^8^ (see also below). Once again, these in vivo findings of increased responsiveness of VMH GI neurons to decreased local glucose level among hyperleptinemic, leptin‐resistant animals are consistent with observations that a) peripheral injection of a low glucose concentration towards the brain increases electrical activity in the nearby arcuate nucleus (also a glucose‐sensing centre) to a greater extent in obese, insulin‐resistant versus normal animals,^23^ (presumably GI neurons though not identified in the study) and b) the normal leptin effect to attenuate the in vitro response of GI neurons to decreases in glucose among healthy animals is reduced in such neurons isolated from insulin‐resistant animals.^36^ However, only the present study simultaneously assesses both VMH GE and GI neuronal electrophysiological activation response to local physiological glucose level increase and decrease, respectively in the intact regular chow vs. obesogenic high‐fat diet fed animal. Consequently, the present study unveils a pathophysiological construct wherein VMH GE neurons of obesity/insulin resistance‐induced HFD versus RC rats would not appropriately sense a meal glucose rise (or GTT glucose rise) resulting in a diminution of the subsequent normal VMH GE neuronal stimulation of insulin sensitivity and peripheral glucose disposal. This VMH neuronal response shift is coupled to an increased sensitivity of VMH GI neurons to hypoglycaemia that would function to maintain an increased level of basal glucose supply to the body/brain. The composite of these two alterations in GE and GI neurons would benefit an animal confronted with a drastic and prolonged ensuing seasonal reduction in environmental food (glucose) supply, providing an evolutionary selection advantage. Indeed, seasonal changes in glucose tolerance are coupled to and driven by seasonal changes in brain dopamine activity (reviewed in refs 6 and 8) that the present study identifies as a significant modulator of the VMH glucose‐sensing system.

While the full scope of neurophysiological modulators of this shift in VMH GE and GI responsiveness to local glucose level changes induced by the HFD insulin‐resistant state remains to be elucidated, the present study focused on the influence of circadian‐timed dopamine agonism in this regard, particularly in relation to noradrenergic input signalling at the VMH. Composite findings from a series of independent studies indicate that a decrease in amplitude of the circadian peak of dopaminergic activity at the SCN potentiates an increase in noradrenergic activity at the VMH which in turn induces the insulin‐resistant, glucose‐intolerant state (1‐6, 8‐10, 37‐41). The present study findings indicate that circadian‐timed bromocriptine administration to mimic (re‐establish) the circadian peak of brain dopamine activity that is attenuated in insulin‐resistant animals including those developed from HFD feeding^3^ normalized both the diminished VMH GE response to increased glucose and the over‐sensitive VMH GI response to glucopenia in HFD‐fed obese animals to resultantly mimic such responses observed in RC‐fed animals (Figure 6). That is, the influence of the HFD to alter the VMH glucose‐sensing apparatus as demonstrated herein to favour the glucose‐intolerant state was reversed by such dopamine agonist treatment even while the animals were maintained on the HFD. Such a dopaminergic effect would be presumed to reduce glucose intolerance and insulin resistance, which it markedly did, in these animals maintained on a HFD (Figure 7).

We next investigated whether or not this circadian‐timed dopaminergic effect on VMH GE glucose sensing may involve its well‐established effect to normalize elevated noradrenergic activity at the VMH in insulin‐resistant animals.^4^, ^11^, ^37^ In insulin‐resistant states, both the presynaptic release of norepinephrine at the VMH and the postsynaptic response to norepinephrine on VMH neurons are elevated^37^, ^38^ and artificial induction of this hyper‐noradrenergic alteration in lean, insulin‐sensitive animals is sufficient to induce insulin resistance and glucose intolerance even in such animals on RC.^9^, ^10^, ^39^, ^40^, ^41^

Bromocriptine treatment has been demonstrated to normalize the elevated noradrenergic activity (its presynaptic release and postsynaptic response both) at the VMH in insulin‐resistant animal models.^4^, ^11^, ^37^ To investigate the possibility that a bromocriptine‐induced decrease of elevated VMH NE activity may participate in the bromocriptine effect to appropriately restore GE neuron sensing of local VMH hyperglycaemia, we investigated if increased VMH NE activity would itself attenuate the response of VMH GE neurons to local hyperglycaemia (at a level associated with postprandial glucose rise, >2.5 mM) in RC‐fed animals. In fact, iontophoretically applied norepinephrine did reduce by 40%‐50% the in vivo responsiveness of VMH GE neurons to local hyperglycaemia. These findings suggest that the VMH increase in NE activity (arising from the brain stem) typically understood as the effector of the VMH GI neuronal activated‐induced counter regulatory response^20^ can also function to reduce the VMH GE response to local hyperglycaemia and thereby block the induction of peripheral insulin action and glucose disposal to potentiate glucose intolerance. Since bromocriptine reduces this increased VMH NE activity and normalizes both the aberrant GE and GI responses to hyperglycaemia and hypoglycaemia, respectively, leading to improved glucose tolerance and insulin sensitivity, the composite of these findings suggests that BC action to normalize elevated VMH NE activity participates in its effect to normalize VMH GE glucose sensing and ultimately reduce glucose intolerance and insulin resistance. It must be appreciated that bromocriptine has been demonstrated to affect several other central pathways that can act to improve glucose intolerance such as reducing elevated VMH serotonin levels, as well as reducing elevated paraventricular nucleus norepinephrine, neuropeptide Y and corticotropin releasing factor levels,^4^, ^11^, ^42^ each of which could contribute to the bromocriptine impact on VMH glucose sensing observed herein, as previously discussed.^6^, ^8^

It is critically noteworthy that BC administered at ZT8 (outside the natural circadian peak window of dopamine at the SCN [and striatum] in healthy glucose tolerant animals)^1^, ^3^ had no effect on reversing the impact of the HFD on VMH GE and GI neuronal glucose sensing. Likewise, the effect of dopamine administered directly to the SCN of insulin‐resistant rats at the time of day of its normal circadian peak at this site in insulin sensitive rats but not outside this circadian interval reversed the insulin‐resistant, glucose‐intolerant state and the elevated VMH norepinephrine level.^3^ That is to say, activation of SCN area neurons in obese HFD‐fed rats by dopamine during the time of day that such dopaminergic activity peaks in healthy insulin‐sensitive animals (but not outside this circadian time window) leads to a decreased release and activity of NE at the VMH that in turn can potentiate or allow for a normalizing shift in GE glucose sensing (as demonstrated herein) to in turn normalize insulin resistance and glucose intolerance. The composite of the present and these past‐related study findings provides a construct wherein animals prone to the obesogenic effects of a HFD reduce brain (SCN) circadian peak dopaminergic activity to elevate NE input to the VMH that in turn inhibits the VMH GE response to physiological glucose rise and stimulates chronic increases in sympathetic drive to adipose, liver, and vasculature to ultimately contribute to insulin resistance syndrome. Whether animals prone to the obesogenic effects of a HFD exhibit the altered responses of VMH GE and GI neurons to hyperglycaemia and hypoglycaemia, respectively, as described herein prior to development of obesity on the HFD was not the aim of this study and cannot be ascertained from the study results. However, with these new data in hand it would now be of interest to determine if the altered VMH GE and GI glucose sensing in obesity‐induced HFD‐fed rats described herein is present in obesogenic prone HFD‐fed animals prior to development of obesity on the HFD (and if so, determine how this neurophysiology differs from HFD‐resistant animals). It is clear from the prodigious work of Levin et al and others^43^, ^44^, ^45^, ^46^, ^47^ that several hypothalamic alterations in key metabolic control pathways are present in animals bred to become HFD obesity prone before development of obesity on the HFD. Interestingly, animals fed an obesogenic HFD exhibit marked reduction in striatal dopamine activity (dopamine release and/or dopamine receptor binding)^48^, ^49^, ^50^, ^51^ (similar to such responses in the SCN^3^), an effect that may involve ingested fatty acid reduction of gut N‐acetyethanolamines, stimulants for striatal dopamine release.^52^

Our previous work indicates that a major site of dopamine neurons projecting circadian dopaminergic input to the SCN that regulates its output control of metabolism arise from the supramammallary nucleus (SuMN).^3^ The SuMN also regulates dopaminergic activity in the striatum which has been implicated in peripheral glucose tolerance regulation.^53^ Moreover, the phase of the circadian dopaminergic input rhythm to the SCN is in phase with the dopamine input rhythm to the striatum.^54^, ^55^ Importantly, decreased dopamine activity at the striatum has been associated with insulin resistance and glucose intolerance in man and pharmacologically inducing a decrease in striatal dopamine levels induces insulin resistance within a day or two in otherwise healthy humans.^56^, ^57^, ^58^ Moreover, dopamine antagonist therapies used as antipsychotic medications all are generally associated with induction of glucose intolerance and weight gain.^59^ The effect of circadian‐timed bromocriptine to normalize VMH glucose sensing in insulin‐resistant animals may involve its action to restore the daily circadian peak in brain dopaminergic activity on striatal neurons as well as such documented effect on the SCN^3^ inasmuch as intracerebroventricular bromocriptine administration or direct SCN dopamine administration each ameliorate glucose intolerance and insulin resistance.^3^, ^12^ It cannot be ruled out however that other central and/or peripheral sites of action of bromocriptine may contribute to its present study effects. Future studies of impact of SCN, striatal, or other site‐specific dopamine receptor activation upon VMH glucose‐sensing neuron activity under different nutritional conditions will be needed to further elucidate this matter. Importantly, the effect of ZT13 circadian‐timed bromocriptine treatment to ameliorate the HFD‐induced diabetogenic shift in VMH glucose sensing was demonstrable at 24 hour after its final day of (a 2 week) treatment. The present observed effect of such bromocriptine treatment may help explain its effect to reduce postprandial glucose without raising plasma insulin levels at the three standard meals of the day in insulin‐resistant and T2DM subjects, even though the agent is removed from the circulation shortly after its early morning administration.^5^, ^6^ Such treatment appears to reprogram the daily central glucose‐sensing response to postprandial glucose across the normal feeding period of the 24‐hour daily cycle.

In summary, relative to rats maintained on regular chow diet, exposure of rats to an obesogenic HFD induces a simultaneous diminution of the VMH GE neuronal response to physiological hyperglycaemia and a increased sensitivity of the VMH GI neuronal response to physiological hypoglycaemia in an in vivo recording system. Circadian‐timed bromocriptine administration at the time of day of the daily peak in central dopaminergic activity in lean insulin‐sensitive animals and that is diminished in obese HFD‐fed animals reverses the effect of the HFD on the VMH GE and GI responses to hyperglycaemia and hypoglycaemia, respectively to mimic conditions in lean insulin‐sensitive animals. This bromocriptine effect is not observed if it is administered outside of this normal central circadian dopamine activity peak window. This effect of appropriately circadian‐timed bromocriptine administration on VMH glucose sensing in obese HFD‐fed animals may in part involve its effect to reduce elevated VMH noradrenergic activity as such VMH activity blocks VMH GE neuronal sensing of hyperglycaemia. This unique effect of circadian‐timed bromocriptine upon VMH glucose‐sensing neurons may explain the ability of similarly circadian‐timed bromocriptine‐QR therapy to reduce postprandial glucose levels across the meals of the day long after the drug is removed from the circulation in type 2 diabetes subjects (reviewed in ref. 6).

## CONFLICT OF INTEREST

All authors are current employees of VeroScience LLC.

## AUTHOR CONTRIBUTIONS

CS, YZ and AC conceived and designed the experiments. CS, YZ and AC performed the experiments. CS, YZ and AC analysed the data. CS, YZ and AC contributed materials. CS, YZ and AC wrote the MS. CS, YZ and AC revised the MS.

## ETHICAL APPROVAL

All experiments were conducted according to policy reviewed by the VeroScience institutional animal care and use committee.

## Supporting information

Supplement Material

## Data Availability

The data that support the findings of this study are available from the corresponding author upon reasonable request.
