# Supplementary material for: Circadian‐timed dopamine agonist treatment reverses high‐fat diet‐induced diabetogenic shift in ventromedial hypothalamic glucose sensing
Source: Endocrinol Diabetes Metab. 2020 May 7;3(3):e00139. doi: 10.1002/edm2.139 (PMC7375120; doi:10.1002/edm2.139)
Supplement: Supplementary file 1 — Supplement Material [file EDM2-3-e00139-s001.docx]

**Supplemental Information: Receiver Operating Characteristic (ROC) Analyses and Results of High Fat Diet Influence on VMH Glucose Sensing**

ROC Test Method

ROC analyses are typically employed in investigations of intervention-induced changes in neuronal activity to assess the probability of such changes to be perceived as true changes by other post-synaptic neurons (or tissues) receiving this new information (e.g. 1). In order to assess if observed changes in multi unit event rate of VMH glucose sensing neurons in response to local hypoglycemia or hyperglycemia were potentially usable by a post-synaptic neuron reading this neural code (a rate based code) (to monitor firing rate within VMH glucose sensing neurons and make biochemical adjustments to reestablish euglycemia in the periphery), we examined the ROC curves for each injection period for both the glucose and 2-DG infusion studies. ROC curves provide a threshold independent analysis of how well moment-to-moment multi-unit firing rate could perform as a discriminator between euglycemic periods (baseline periods) from periods that were either hyperglycemic or hypoglycemic induced by local glucose or 2-DG infusion, respectively).

ROC curves were calculated for both glucose and 2-DG injection experiments at each dosage level tested. For each intracranial injection dose of glucose or 2-DG the average multi-unit event rate calculated as the number of detected action potentials within a one second window was obtained. An equal number of time windows were selected from the 60 second baseline recording period which preceded each injection. Each observed firing rate from the collection of 120 one-second bins from a recording was independently tested as a threshold discriminator to accurately predict whether that firing rate was elicited from the baseline or stimulated condition.

That is, each value from the family of available criterion response levels (e.g., 1,3,5 spikes/sec at baseline or 17,21, 29 spikes/sec during stimulation) was selected one by one and tested, with the proportion of time windows from baseline recording periods with firing rates equal or higher than the current tested criterion level representing false positives, and the proportion of time windows from injection periods with firing rates at or above the current tested criterion level representing true positives. The true positive rate and false positive rates were calculated for each of the values found in the family of criterion response levels. The ROC curve manifests the ability of each independent criterion response level to accurately discriminate baseline periods from injection periods of either glucose or 2-DG and was assessed by calculating the true positive rate (y-axis, an index of “sensitivity”) and false positive rate (x-axis, “1 − specificity”), at each and every possible threshold level by sampling each criterion response level. Performance of multi-unit event rate as a discriminator was quantified by calculating the area under the curve (AUC) of the ROC curves. ROC curves and the respective AUC values were calculated for each dose of elevated glucose (3 – 5 mM) and each dose of 2-DG (1 -15 mM) at the VMH.

ROC Test Results and Discussion

In order to determine if these changes in multi-unit response rate could be functionally relevant, receiver-operator-characteristic (ROC) curves were calculated for each infusion. Multi-unit activity rates were calculated for each 1 second period of baseline recording and during each 1 second period during drug infusion. For each curve the area under the curve (AUC) was determined to quantify signal detectably. Example ROC curves from 5mM glucose infusions into rats on regular diet and high fat diet can be seen in supplemental figure 1A, and similar ROC curves for 1-15 mM 2-DG infusions can be seen in supplemental figure 1B.

The dose response curve of AUC values from ROC curves from Glucose infusions of 3, 4 and 5 mM glucose can be seen in supplemental figure 1C. There was a significant main effect of glucose dose on ROC AUC values (F = 7.91, p<0.05). Additionally, there was a significant main affect of treatment on AUC values for glucose infusions (ANOVA, F = 7.734, p<0.01), however, while no significant interaction between dose and diet group was observed (ANOVA, F = 1.610, p = 0.24). Post-hoc analysis (Bonferroni) revealed that the two curves departed significantly at only the highest dose (p < 0.01).

The dose response curve of AUC values from ROC curves from 2-DG infusions can be seen in supplemental figure 1D. There was a significant main effect of dose on ROC AUC values (ANOVA, F = 14.82, p <0.001) and a significant main affect of diet such that AUC values were higher for ROC curves obtained from animals maintained on a high fat diet (ANOVA, F = 5.23, p<0.05), while no significant interaction between dose and diet group was seen (ANOVA, F = 0.34, p=0.846).


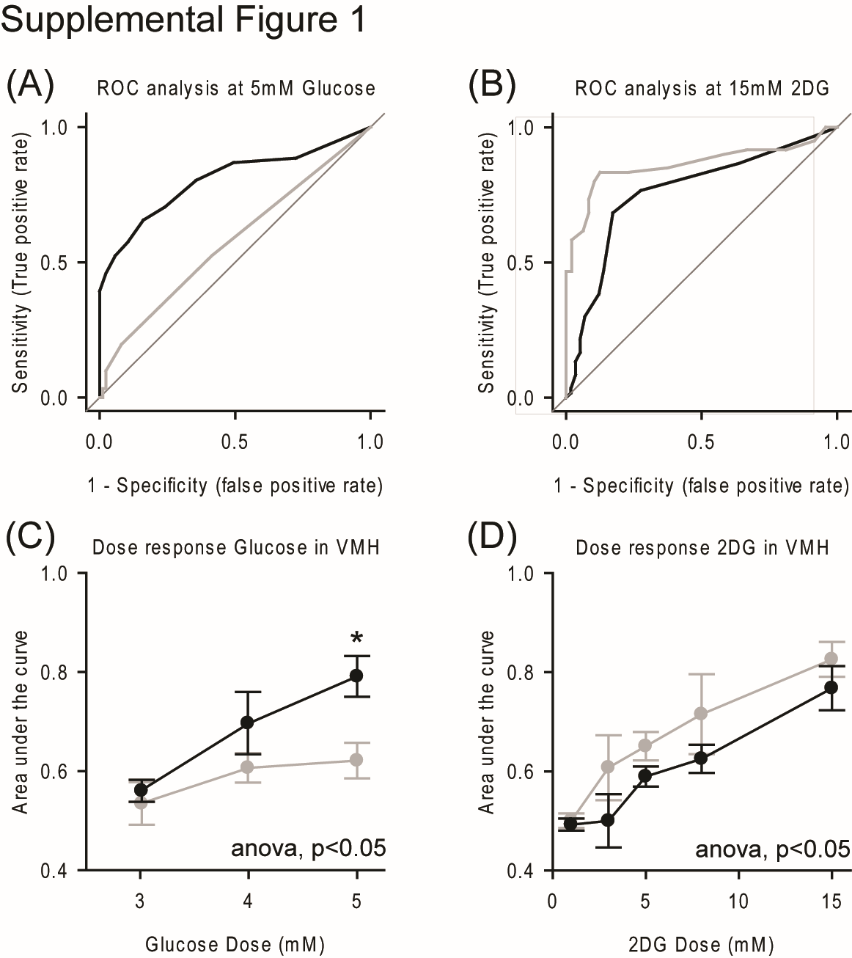


**Supplemental figure 1. Diet induced changes in fuel sensing of VMH neurons impact signal detectability of VMH output.** A) ROC curves obtained from spontaneous activity from VMH neurons and during local injection of 3-5mM glucose among rats maintained on a regular diet (black line) or a high fat diet (dashed line). B) ROC curves obtained from spontaneous activity from VMH neurons and during local injection of 1-15mM 2-deoxyglucose (2-DG) from rats maintained on a regular diet (black line) or a high fat diet (dashed line). ROC curves were calculated for all doses across all conditions, and quantified by calculating the area under the curve (AUC) for each. C) Dose response curve of AUC for 3, 4 and 5 mM doses of locally infused glucose. D) Dose response curve of AUC for all doses (1-15mM) of locally infused 2-DG.

Both VMH glucose excited (GE) and glucose inhibited (GI) neurons contribute to the excitatory population recordings presented here. While it is possible that these two groups of neurons target different populations, the exact specificity of their projections, and the underlying neural code used by these VMH neurons are still uncertain. For that reason it is important to establish if effect sizes following drug infusion of glucose of 2-Deoxyglucose could be detected by an idealized observer of that signal (any post-synaptic neuron) to accurately determine periods of low (e.g., fasting) or high (e.g., feeding) brain (and peripheral) glucose level, given the inherent variability of spontaneous firing of these neurons. In animals with a history of high fat diet feeding, the reduced area under the curve from the glucose injection ROC data suggest that (post-synaptic) detectability of VMH communication of high brain and peripheral glucose levels is significantly impaired, while the increased AUC from the 2-DG ROC curves suggest an inappropriate elevated detectability of VMH communication of local hypoglycemia. These findings reaffirm the results of the time-point dose response analyses of VMH GE and GI neurons to hyperglycemia and hypoglycemia, respectively, respecting influence of high fat diet feeding on both hyperglycemia and hypoglycemia sensing. Collectively, the findings of the present study strongly suggest that high fat diet feeding alters VMH GE and GI neurons in a manner that inappropriately sense glucose levels and transmit this information to potentiate hyperglycemia during fasting and feeding periods of the day.

REFERENCE

1. Stoelzel CR, Huff JM, Bereshpolova Y, Zhuang J, Hei X, Alonso JM, et al. Hour-long adaptation in the awake early visual system. J Neurophysiol. 2015;114(2):1172-82.
